# Adopted transgender subjects are overrepresented and have a different psychosocial profile than their non-adopted counterparts: A case-control study

**DOI:** 10.1371/journal.pone.0322201

**Published:** 2025-04-29

**Authors:** Iris Yaish, Yona Greenman, Karen M. Tordjman

**Affiliations:** 1 The Transgender Health Center, Institute of Endocrinology, Metabolism, and Hypertension, Tel Aviv Sourasky Medical Center, Tel Aviv, Israel; 2 Faculty of Medicine, Tel Aviv University, Tel Aviv, Israel; Amity University, INDIA

## Abstract

**Background:**

The factors driving the global increase in the number of transgender individuals remain unclear. It has been suggested that early-life events may interact with biological predispositions, yet little research has explored specific early-life circumstances that might contribute to gender incongruence. One such factor is adoption, as a few pediatric gender identity clinics have reported an overrepresentation of adoptees. However, no study has systematically examined whether this trend extends to adult transgender health centers, nor has the psychosocial profile of adopted transgender individuals been characterized in adulthood.

**Aims and methods:**

This retrospective study was conducted in two phases to evaluate the prevalence and characteristics of adoptees among adult transgender patients seeking gender-affirming therapy. In Phase 1, we analyzed a cohort of 671 new adult subjects presenting between 2015 and 2021. In Phase 2, the nested case-control analysis, the 15 adoptees identified were matched in a 1:4 ratio with non-adopted controls based on age, assigned sex at birth, and presentation timing. Data on demographics, psychosocial factors, psychiatric diagnoses, and parental support were extracted from electronic records, and augmented by telephone interviews when needed.

**Results:**

Adoptees constituted 2.2% of the clinic population, an order of magnitude higher than the national rate (P < 0.0001), with an unprecedented assigned-female-at-birth (AFAB) ratio of 73.3%. After matching, 60% of adoptees had at least one psychiatric co-morbidity, almost twice the rate of non-adopted controls (OR = 3.23, 95% CI: 1.02–10.21, P = 0.042). Despite coming from higher socioeconomic status homes (P < 0.001), adoptees had lower odds of achieving college education (P = 0.031), and receiving full parental support for transition (OR = 0.20, 95% CI: 0.05–0.71, P = 0.015). Notably, 28.6% of adoptees had attempted suicide vs 3.3% of non-adopted controls (OR = 11.6, 95% CI: 1.87–71.97, P = 0.01).

**Conclusions:**

Adopted transgender individuals represent a vulnerable subgroup within the transgender population, characterized by unique psychosocial challenges. Our findings underscore the importance of tailored interventions and heightened support within transgender health clinics for adoptees seeking gender-affirming therapy. Further research is warranted to elucidate the interplay of adoption, biological predispositions, and social factors in the development of gender incongruence.

## Introduction

The increased visibility of the transgender community worldwide is not only due to the readiness of the media and that of the various social platforms to report on it, or to give it access, it is truly the manifestation of a major and global social shift [1].

Data originating from medical centers across the world that provide gender-affirming treatment, suggest that in the last two decade these numbers have increased by five to 10-fold in parallel with the inflation in scientific publications in the field [2–4]. Although precise numbers are difficult to come by, the recent US Census Bureau Survey estimates that about one percent of the adult US population, or roughly 2.6 million people, self-identify as transgender [5]. Furthermore, there is evidence that the sharpest increase stems from youths whose relative representation within the transgender community has almost doubled in less than five years [6].

Gender incongruence, as it has come to be known [7], and its underlying basis are still challenging researchers who lack a unifying explanation. Many transgender subjects now present in their youths, while a large fraction of those who present in adulthood report having experienced a different gender identity than their at-birth assigned sex since childhood [8], suggesting an early onset of gender incongruence. While there is no definite mechanism, theories have been put forth based on various observations. Proponents of a biological basis have suggested exposure to high in utero levels of androgens as a potential contributor to male-like identity in assigned-female-at-birth (AFAB) persons. Given the fast-growing numbers, it stands to reason that no rapidly emerging mutation can explain it. However, a contributing genetic basis has been deemed possible as rare genetic variants [9], and epigenetic changes have been reported in genes associated with sex-steroid-related brain plasticity [10]. Subtle structural and connectivity brain changes have likewise been documented in transgender individuals. It has been also been suggested that gender incongruence could stem from multi-faceted social and developmental issues that interact, early in life, with the right biological background. One such event could be adoption in infancy as it has been shown that early deprivation among adoptees was accompanied by lasting epigenetic changes and increased DNA methylation [11]. Adoptees have also been reported to have a slightly higher prevalence of certain psychiatric conditions compared to their non-adopted peers, though the overall impact remains modest [12–14]. These findings suggest that early-life factors may contribute to psychological vulnerabilities in some adoptees, though the extent to which this influences identity development remains unclear.

Overrepresentation of adoptees among subjects in identity disorder clinics has been the object a of a few reports. As early as 1998, a study conducted in a children gender identity clinic by Zucker and Bradley reported that male subjects adopted at an early age comprised 7.6% of their clinic’s population, while the prevalence of adoptions in Ontario at the time was only 1.49% [15]. Similar observations emanated subsequently from other pediatric gender identity clinics [16,17]. However, to the best of our knowledge, this issue has never been looked at in the context of a contemporary large cohort of adult patients receiving gender-affirming hormone therapy (GAHT) for gender incongruence.

As we were likewise struck by an apparent overrepresentation of adoptees among subjects attending our clinic, we sought to precisely assess this question among our patients, and to determine if there were characteristics that differentiated adoptees from non-adopted subjects.

## Methods

### Methods study design and population

This retrospective case-control study was conducted in two phases. First, we analyzed the cohort of 671 new adult subjects (age ≥ 18) who presented to our Transgender Heath Center seeking gender-affirming hormone therapy (GAHT) between January 1, 2015, and December 31, 2021. Second, we performed a nested case-control analysis of identified adoptees and matched controls.

The matching procedure was as follows.

All 15 adoptees identified in the cohort were included as cases. For each adopted subject, we selected four non-adopted controls using the following matching criteria: - Same sex assigned at birth (SAB); Age within one year of the adopted subject; Initial presentation within the same six-month period. Controls were selected chronologically from all eligible matches, without knowledge of their psychosocial characteristics or outcomes.

Data were extracted from the subjects’ electronic records, and augmented when needed by telephone contact. Quality assurance measures included the use of standardized data extraction forms, double-checking of all entered data, validation of unclear or missing information through telephone interviews, and documentation of data completeness for each variable.

A waiver for informed consent was granted for anonymized data extraction from electronic medical records, while the study received approval for telephone consent for additional information deemed necessary to investigators (Tel Aviv Sourasky Medical Center Institutional Review Board approval #TLV-0434-21).

### Data collection and privacy protection

Data included demographics, marital status, ethnic background, age at onset of dysphoria, education level, professional qualifications, employment status, history of military service, psychosocial assessment and psychiatric diagnoses if any (every subject is required to provide a psychosocial assessment as a prerequisite for GAHT initiation), and medical background. Subjective socioeconomic status (SES) was determined using the extensively validated 10 rung MacArthur scale [18,19]. It was further narrowed down to 3 categories: low (rungs 1–3), middle (4–7), and high (8–10) [20]. Information was also collected regarding the level of support each subject received from his/her family and social circle regarding the transition process. All these data are, for the most part, routinely collected at the time of intake of any new transgender patient, and are included in the electronic medical records as “confidential data” inaccessible to unauthorized personnel. For a small portion of cases (less than 10%), we conducted phone calls to gather missing information from the charts. This primarily involved details about education level, employment status, and living arrangements (whether in the parental home or independently). All subjects we reached by phone willingly provided the requested information. Adoptees-specific information included age at adoption, country of origin (a substantial fraction of adoptees in Israel were born in foreign countries), age and gender of adoptive parents.

Data collection was completed by the P.I (I.Y) between 11.04.2021 and 11.02.2022. All data were anonymized, and none of the co-authors had any access to identifiers while handling the data.

### Statistics

The only continuous variable was age, this is presented as median and interquartile range (IQR), the Mann Whitney test was used for age comparisons. All other variables were categorical, and are therefore given as proportions or percentages.

The rate of adoptees in our clinic population was first compared to that in the general Israeli population, as computed from the 2018 data published by the Central Bureau of Statistics [21,22] by the one-sample binomial test. We next compared the general characteristics (age at presentation, and sex-assigned-at birth, SAB) of adopted subjects to those of non-adopted subjects in our cohort. After the matching procedure, all data retrieved for both cases and controls were compared. Regression-based imputation was utilized to handle variables with missing data in over 5% of cases. All categorical variables were compared by the chi-squared test for proportions or the Fisher’s exact test. For all major outcomes. Odds ratios (OR) with their 95% confidence intervals (CI) were calculated. Correlations between variables were analyzed by the Spearman’s correlation coefficient. All statistical computations were performed with SPSS 29.0 (IBM Corp Armonk, NY). Statistical significance was assumed for P < 0.05

## Results

### Phase 1: Analysis of the entire clinic cohort

#### Rate of adopted patients in the clinic population.

The entire cohort of subjects presenting for the first time to the clinic during the study period consisted of 671 people. Of those, 15 were adopted, or a rate of 2.2%. This is roughly an order of magnitude higher than the 0.22% rate of adoptees in the Israeli population during the corresponding period (P < 0.0001) [21,22].

#### Comparison of adoptees to the general clinic population.

While the median age at the first visit of adoptees and non-adopted subjects was identical 23.0 y [19–30], the distribution of sex assigned-at-birth (SAB) was different. Of the 15 adoptees, only 4 (26.7%) were assigned-male-at-birth (AMAB), while the SAB was female (assigned female-at-birth-AFAB) for the other 11. This is in contrast with the 656 non-adopted subjects in the entire cohort of new patients during the study period, in whom the SAB distribution was fairly balanced, AMAB in 322 cases (49.1%), and AFAB in 334 (50.9%), P = 0.085

### Phase 2: Nested case-control analysis

#### Characteristics of the adoptee cohort.

**Fig 1:**
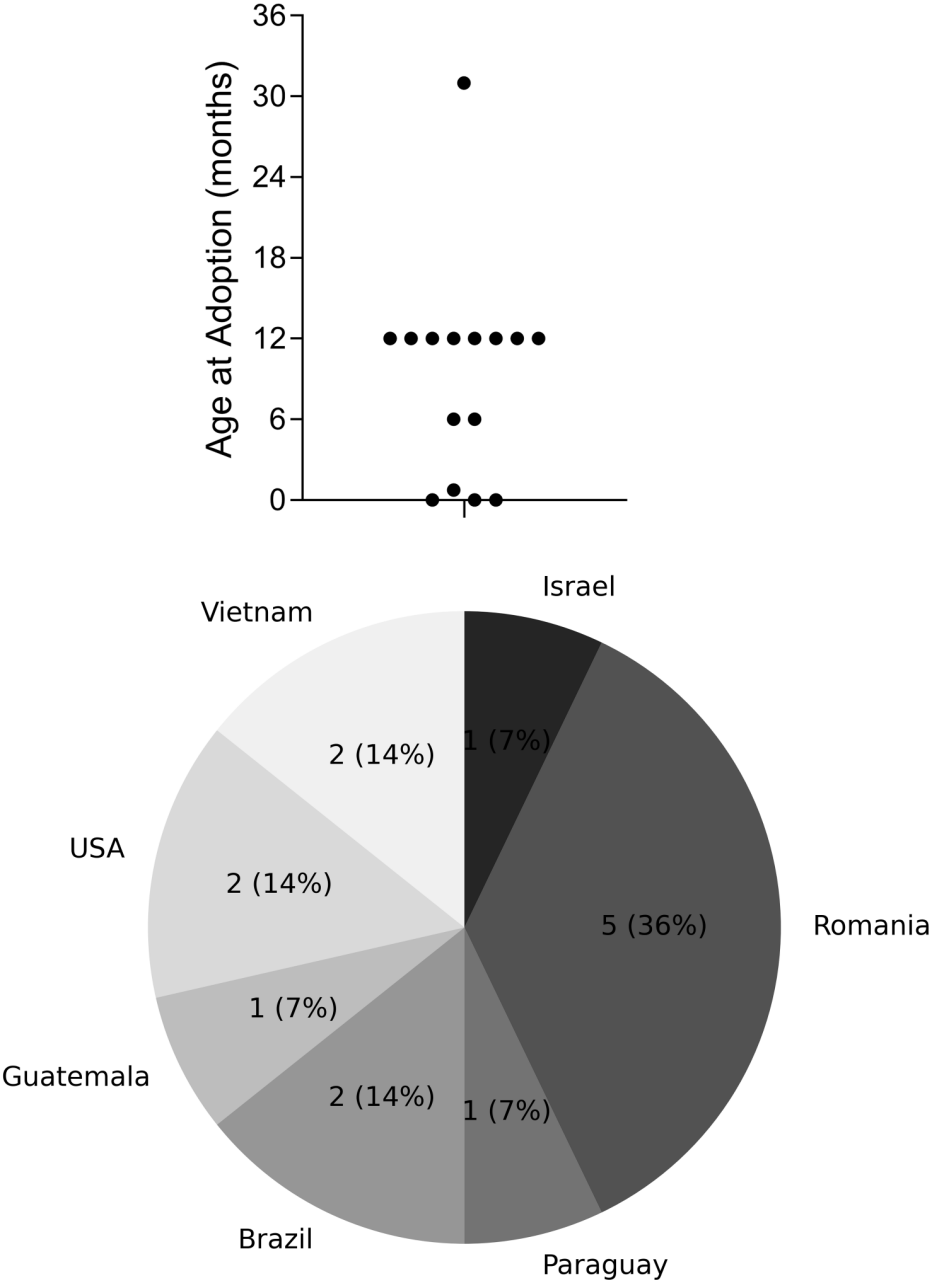
Demographic data of subjects at the time of adoption. a. Distribution of age at the time of adoption in months; b. Country of birth.

Age at adoption ranged from birth to 32 months, with a mean of 9.5 months, a median and a mode of 12 months. The age distribution at the time of adoption and country of origin are shown in Fig 1a and b. With the exception of one person, all subjects had been adopted before or at the age of 12 months. While adoptive parents were mostly from Israel themselves, the international adoption pattern, that was common two to three decades ago, is visible. Only one subject was born in Israel, while Romania was the most common country of adoption. As is often the case [23], adoptive parents were rather elderly, with a median of 40 years [IQR 34.5–43.5] at the time of adoption, the youngest parent was 24 years old and the oldest 50. One subject had been raised by a single mother. Parents divorced after the adoption in two cases.

#### Demographics of adopted and non-adopted subjects after matching.

As we corrected for possible SAB imbalance by matching, age and SAB were now similar in both groups, Table 1.

**Table 1:** Demographics and socioeconomic data of adopted and non-adopted controls at the time of presentation.

Variable	Adopted N = 15 (%)	Non-adopted N = 60 (%)	P
**Age at 1**^**st**^ **visit (y)** *Median [IQR]*	23.0 [19.0–30.0]	22 [20.0–28.0]	0.867
**AFAB-*n (%)***	11 (73.3)	44 (73.3)	1.0
**Marital status*-n (%)***			
*Single*	12 (80.0)	44 (81.5)	0.428
*Married*	2 (13.3)	3 (5.6)	
*In a relationship*	1 (6.7)	7 (13.0)	
*Missing*	N/A	6 (10.0)	
**Education-n (%)**			
*Elementary*	3 (20.0)	5 (8.8)	0.048
*High school*	12 (80.0)	36 (63.2)	
*College*	0	16 (28.1)	
*Missing*	N/A	3 (5.0)	
**College education**			
*Yes/no (n/n)*	0/15	16/41	0.031
*Missing*	N/A	3 (5.0)	
**Military/civil service**			
*Yes/no*	3/12	28/25	0.086
*Missing*	N/A	7 (11.7)	
**Employment**			
*Yes/no (n/n)*	9/6	43/13	0.206
*Missing*	N/A	4 (5.0)	
**SES-*n (%)***			
*Low*	0 (0)	11 (18.3)	<0.001
*Medium*	11 (73.3)	29 (48.3)	
*High*	4 (26.7)	0 (0)	
*Unknown*	N/A	20 (33.3)	

AFAB: Assigned-female-at-birth; SES: Subjective socioeconomic status

When questioned about the precise age of dysphoria onset, an item we could obtain a response on from only 39 subjects, adopted subjects recalled experiencing dysphoria at a younger median age of 10 y [IQR 5.5–12.5] vs. 14 [12.0–16.0], P = 0.007. However, almost all subjects could respond whether dysphoria had started in childhood (before the age of 12 y) When analyzed by categories, this difference disappeared as 78.6% of adoptees recalled having experienced the onset of dysphoria in childhood, compared to 64.8% of non-adopted subjects, P = 0.523.

There was no difference in marital status between the groups, as a vast majority (over 3/4) of these young subjects were single. However, other traits of “social conformity” such as acquiring a higher education, having served a period of military or civil community service (in lieu of military service), an accepted index of healthy social functioning in the Israeli society [24], were either significantly higher, or tended to be so among the non-adopted controls. A finding which appears to contrast with the significantly higher subjective SES of adopted subjects. Indeed, almost a third of adoptees reported coming from high SES families, as opposed to none of the non-adopted controls (P < 0.001). Although employment status was not significantly different between the groups, possibly due to the limited numbers, it is still noteworthy that 76% of the non-adopted subjects were gainfully employed as opposed to only 57% of the adoptees (P = 0.145).

#### Psychiatric comorbidities, suicide attempts, and substance abuse.

It has been extensively documented that psychiatric comorbidities are prevalent among the transgender community [25,26].

Therefore, as per our center protocol, all transgender patients desirous to receive GAHT are requested to undergo a formal psychosocial assessment before their first visit, the results of which may alter the treatment plan. In the entire cohort the prevalence of any psychiatric co-morbidity was recorded in 37.3% of subjects (28/75). The prevalence of any psychiatric diagnosis among adoptees was about twice as high than among non-adopted controls, 60% vs. 31.7%, (OR = 3.23, CI: 1.02–10.21, P = 0.042).

The distribution of the various psychiatric diagnoses within each group of subjects is presented in Table 2. The most common diagnoses were depression and/or anxiety, that were present in 26.7% of the entire cohort (20/75), but they were equally prevalent in both groups. Nonetheless, adoptees were significantly more likely to have attempted suicide as 28.6% of them had, as opposed to only 3.3% of non-adopted subjects, (OR = 11.6, 95% CI: 1.87–71.97, P = 0.01)

**Table 2:** Psychiatric comorbidities, lifestyle habits, and risk behaviors in adopted versus non-adopted transgender individuals. Variable frequencies are presented as number of subjects with/without the condition in each group. OR: Odds Ratio; CI: Confidence Interval; NC: Not calculable due to zero cells. In a few cases the information was missing, this is reflected by the numbers not adding to the entire group size.

Condition	Adopted N = 15	Non-adopted N = 60	OR	CI	P
**Any diagnosis**	9/6	19/41	3.23	1.02–10.21	0.042
**Depression**	5/10	12/48	2.0	0.58–6.89	0.308
**Anxiety**	3/12	6/54	2.25	0.49–10.33	0.372
**Bipolar disorder**	0/15	1/59	NC		1.0
**Autism**	0/15	2/58	NC		1.0
**ADHD**	2/13	3/57	2.92	0.44–19.41	0.26
**OCD**	0/15	2/58	NC		1.0
**PTSD**	1/14	3/57	1.36	0.13–14.02	1.0
**Borderline personality**	2/13	2/58	4.46	0.57–34.84	0.176
**Eating disorder**	0/15	2/58	NC		1.0
**Suicide attempt**	4/10	2/58	11.6	1.87–71.97	0.01
**Smoking**	9/6	11/49	6.68	1.98–22.54	0.002
**Cannabis**	4/11	1/56	20.36	2.08–199.3	0.006
**Alcohol abuse**	2/13	1/58	8.92	0.75–106.2	0.103
**Physical activity**	6/9	27/30	0.74	0.24–2.31	0.773

ADHD: Attention deficit hyperactivity disorder; OCD: Obsessive compulsive disorder; PTSD: Post-traumatic stress disorder

The groups also differed with respect to lifestyle and substance abuse. Cigarette smoking was more prevalent among adoptees as 60% of them smoked, as opposed to only 18.3% of non-adopted subjects (OR = 6.68, 95% CI: 1.98–22.54, P = 0.002). Although none of the subjects in the cohort reported illegal hard drug abuse, cannabis consumption was significantly more common among adoptees, 26.7% versus 1.7% (OR = 20.36, 95% CI: 2.08–199.3, P = 0.006).

#### Family support and living arrangements.

Adopted subjects seemed to enjoy somewhat less parental support in their transition process as only 53.3% of them reported full support (from both parents), while 85.4% of the non-adopted control subjects had full parental support, (OR = 0.20, 95% CI: 0.05–0.71, P = 0.015). This difference coincided with a significant greater proportion of adoptees who had left the parental domicile. Only 4/15 (26.7%) of the adoptees were living with their parents at the time of the first visit, whereas 28 of the 46 non-adopted controls (60.9%) for whom the information was available cited their parents’ home as their own, (OR = 0.23, 95% CI: 0.07–0.82, P = 0.036). In fact, there was a correlation between enjoying full parental support for the transition and still living with parents at the time of referral, r = 0.432, P < 0.001. In contrast, not living with parents was not correlated with age at the time of the first visit. Finally, lack of parental support was not associated with a history of suicide attempt.

## Discussion

In this survey, we confirmed for the first time in an adult transgender health center, the overrepresentation of adoptees previously reported among pediatric transgender subjects [15–17]. This overrepresentation in our recent cohort amounts to an order of magnitude greater than the prevalence of adoption in the Israeli population. In their 2017 report Shumer et al. identified 15 adopted individuals among the 184 pediatric subjects who had presented at their program between 2007 and 2015 [17]. However, the overrepresentation of adoptees in their cohort only amounted only to 3.4 time the national rate. Rates of adoptions in Israel, a nation that values procreation, are extremely low, possibly due to the generous and unlimited healthcare support for assisted reproduction and particularly IVF until a woman turns 45 [27]. It is thus not surprising that adoptive parents in this sample were elderly, as they usually resorted to adoption after failing other means. Unfortunately, the age of adoptive parents was not given in the Shumer’s report.

In the Shumer’s study, adoptees were significantly younger than non-adopted patients, whereas in this cohort, composed of largely young adults, age was by design identical between the two groups (23.0; IQR 19–30 y). Moreover, contrarily to the Shumer’s report where the SAB ratio was balanced, we found a very significant preponderance of AFAB (almost 3/4 of all adoptees were born females). Although a growing fraction of AFAB individuals is now being reported, particularly from pediatric transgender clinics [2,28], to the best of our knowledge, such a SAB imbalance has never been reported before.

Therefore, we concluded that implementing a matching procedure was necessary to more accurately characterize the transgender individuals adopted within our cohort. Upon completion of this process, distinct characteristics emerged, setting the adopted individuals apart from the non-adopted controls. Notably, adopted subjects came predominantly from families with higher SES. This observation itself came as no surprise as it concurs with existing research, which indicates a bias towards higher SES among adoptive families, as adoptive parents need to have the means to overcome the bureaucratic and financial challenges of adoption. A number of studies have consistently shown that children from higher SES backgrounds tend to achieve better academic outcomes. Even within the adoptee population, significant emphasis has been placed on the influence of adoptive parents’ SES and educational attainment on the academic and social development of adoptees [29]. Therefore, it is noteworthy that the level of educational achievement among adoptees was significantly lower compared to their non-adopted counterparts. Particularly striking was the absence of college education among adoptees, contrasting with 28% of their age-matched non-adopted counterparts who had attained this level of education.

Military service is mandatory in Israel for both men and women upon reaching the age of 18, with exemptions granted for medical, religious, or ethnic reasons (notably, the Arab Israeli population is largely exempt). Serving in the military or engaging instead in community service is considered a fundamental duty in the Israeli society, and individuals who avoid the draft often face societal disapproval [24]. Over the past decade, the Israeli military has made significant strides in accommodating transgender soldiers by providing trans-friendly facilities such as dedicated bathrooms and individual lodging, offering the whole gamut of gender-affirming medical procedures, and ensuring access to psychological support. Consequently, being transgender does not automatically exempt individuals from military service. Given this context, the fact that 53% of non-adopted subjects fulfilled their military service obligation, compared to only 20% of adoptees, is particularly noteworthy.

The primary focus of the disparity between adoptees and non-adopted controls revolved around the psychosocial profiles of the individuals. Psychiatric co-morbidities are commonly observed in the transgender population, with prevalence rates varying widely across different studies depending on factors such as country of origin and study period. We chose to gauge our present findings in the light of a previous survey from our clinic, the only one coming out of Israel so far, that focused on the prevalence of psychiatric co-morbidities in our transgender clinic population. This study conducted between 2000 and 2018, encompassed 405 subjects and revealed that 35.8% of them carried at least one psychiatric diagnosis [30]. This is almost identical to what was found in this selected, and somewhat more recent cohort, where 37.3% of all subjects had received at least one psychiatric diagnosis. However, when analyzed by adoption status, a large disparity emerged as adoptees were almost twice as likely to suffer from at least one psychiatric condition than non-adopted controls.

The most concerning aspect of these disparities was the prevalence of attempted suicide prior to the first clinic visit. In our earlier study, we were struck by the relatively low incidence of suicide attempts in our cohort, especially when compared to data from other countries [31]. However, the rate we have now uncovered among adoptees ranks among some of the highest reported globally. While this finding is striking, the wide confidence intervals in our findings - a natural consequence of our relatively small sample size - suggest caution in interpreting the precise magnitude of these differences. Nevertheless, the consistent pattern of higher odds ratios across multiple psychiatric and behavioral measures, including substance use, suggests a genuine phenomenon of increased vulnerability in this population.

Furthermore, to underscore the vulnerable psychosocial profile of adopted transgender individuals, we also found evidence indicating significantly lower parental support during their transition process. This was reflected in the fact that only 26.7% of adoptees were still residing with their parents, in stark contrast to 60.9% of non-adopted controls. This discrepancy is noteworthy, particularly when considering that, according to recent data from the Israeli Central Bureau of Statistics, 50% of young Israeli adults aged 18–34 still live with their parents [32].

This study has several strengths. This recent cohort was carefully assembled within a limited timeframe, ensuring consistency in the evaluation and treatment protocols applied at our center. The meticulous matching procedure allowed us to mitigate potential confounders and concentrate on analyzing the primary outcomes. We are particularly confident in the internal validity of the psychiatric assessment, as our current findings are consistent with those of our earlier cohort [30]. In addition, as all subjects were adults at the time of referral, but had been overwhelmingly experiencing dysphoria in childhood, we are also confident we are not dealing with the disputed entity of rapid onset gender dysphoria [33].

However, like any retrospective study, this investigation has its limitations. While the psychiatric evaluation, overseen by mental health professionals, maintains high reliability, we encountered missing data in the control population, such as parental age and education level, as these details are not routinely recorded. Similarly, information regarding the number of siblings for both adoptees and non-adopted individuals was limited. Additionally, there might have been a bias on the part of admitting physicians, who were possibly more meticulous in documenting the psychiatric history of adoptees, particularly regarding suicide attempts.

Again, another limitation is our relatively small sample size, particularly in the adoptee group, which resulted in wide confidence intervals for some of our effect estimates. While this limits our ability to precisely quantify the magnitude of some associations, it does not negate the consistent pattern of increased vulnerability we observed across multiple measures.

While the precise origins of a transgender identity remain unclear, the various proposed mechanisms for triggering gender incongruence cannot be refuted by the current data. Nevertheless, although causality cannot be inferred, the association between adoption and the onset of gender incongruence in childhood is intriguing and warrants further investigation. In such research endeavors, the striking imbalance favoring AFAB individuals documented here should be taken into account.

In conclusion, despite the limitations of our sample size, our findings consistently suggest that adopted individuals are not only disproportionately represented in the transgender population, but they also constitute a highly vulnerable subgroup. Therefore, we recommend that transgender health clinics prioritize adoptees seeking gender-affirming therapy, leveraging all available psychosocial resources while helping these subjects in their transition.
